# Poly[μ_2_-aqua-bis­[μ_4_-2-(1*H*-1,2,3-benzotriazol-1-yl)acetato]­dipotassium]

**DOI:** 10.1107/S1600536812008963

**Published:** 2012-03-07

**Authors:** Qiong Liu

**Affiliations:** a Department of Environment Engineering and Chemistry, Luoyang Institute of Science and Technology, 471023 Luoyang, People’s Republic of China

## Abstract

In the title compound, [K_2_(C_8_H_6_N_3_O_2_)_2_(H_2_O)]_*n*_, each K^+^ ion is seven-coordinated by one O atom from a bridging water mol­ecule, five carboxyl­ate O atoms and one N atom from a benzotriazole group, forming a distorted mono-capped octa­hedral geometry. In the crystal, the carboxyl­ate groups act as bridging ligands, forming a two-dimensional polymer parallel to (001). The aqua ligand, which lies on a twofold rotation axis, forms inter­molecular O—H⋯O hydrogen bonds within these layers.

## Related literature

For background and the synthesis, see: Hu *et al.* (2008 ▶). 
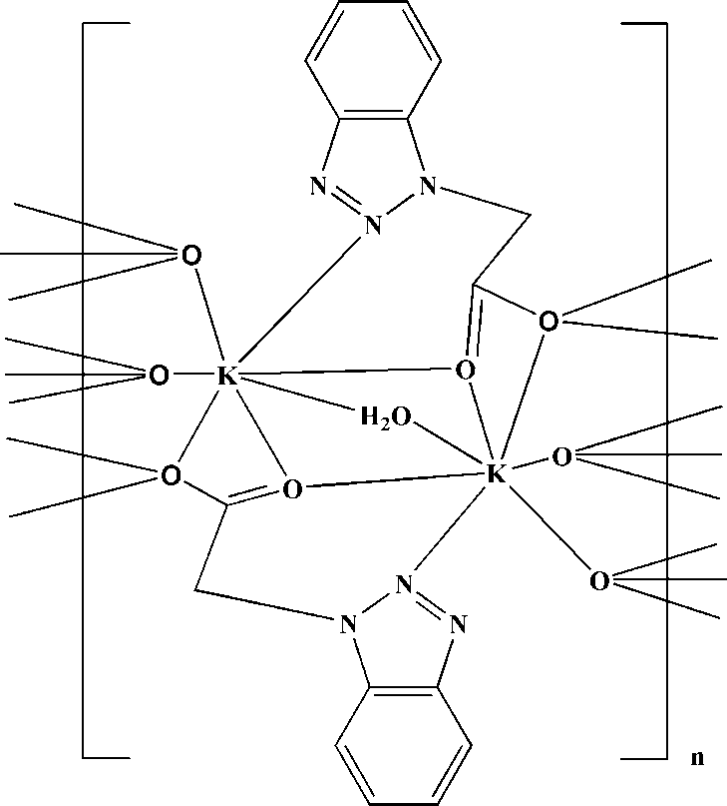


## Experimental

### 

#### Crystal data

[K_2_(C_8_H_6_N_3_O_2_)_2_(H_2_O)]
*M* *_r_* = 448.53
Monoclinic, 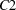

*a* = 12.159 (2) Å
*b* = 4.5893 (9) Å
*c* = 17.666 (4) Å
β = 104.98 (3)°
*V* = 952.2 (3) Å^3^
*Z* = 2
Mo *K*α radiation
μ = 0.54 mm^−1^
*T* = 296 K
0.30 × 0.20 × 0.12 mm

#### Data collection

Bruker SMART CCD area-detector diffractometer
Absorption correction: multi-scan (*SADABS*; Sheldrick, 1996 ▶) *T*_min_ = 0.878, *T*_max_ = 0.937
4827 measured reflections
2140 independent reflections
1508 reflections with *I* > 2σ(*I*)
*R*_int_ = 0.053

#### Refinement

*R*[*F* ^2^ > 2σ(*F* ^2^)] = 0.069
*wR*(*F* ^2^) = 0.135
*S* = 1.05
2140 reflections
136 parameters
H atoms treated by a mixture of independent and constrained refinement
Δρ_max_ = 0.46 e Å^−3^
Δρ_min_ = −0.24 e Å^−3^
Absolute structure: Flack (1983 ▶), 925 Friedel pairs
Flack parameter: −0.02 (9)

### 

Data collection: *SMART* (Bruker, 2007 ▶); cell refinement: *SAINT* (Bruker, 2007 ▶); data reduction: *SAINT*; program(s) used to solve structure: *SHELXS97* (Sheldrick, 2008 ▶); program(s) used to refine structure: *SHELXL97* (Sheldrick, 2008 ▶); molecular graphics: *SHELXTL* (Sheldrick, 2008 ▶); software used to prepare material for publication: *SHELXTL*.

## Supplementary Material

Crystal structure: contains datablock(s) global, I. DOI: 10.1107/S1600536812008963/lh5422sup1.cif

Structure factors: contains datablock(s) I. DOI: 10.1107/S1600536812008963/lh5422Isup2.hkl

Additional supplementary materials:  crystallographic information; 3D view; checkCIF report

## Figures and Tables

**Table 1:** Selected bond lengths (Å)

K1—O2^i^	2.718 (4)
K1—O3	2.760 (3)
K1—O2^ii^	2.829 (3)
K1—O2^iii^	2.835 (4)
K1—O1	2.872 (4)
K1—N2	2.934 (4)
K1—O1^ii^	3.287 (3)

Symmetry codes: (i) 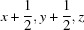; (ii) 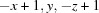; (iii) 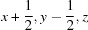.

**Table 2:** Hydrogen-bond geometry (Å, °)

*D*—H⋯*A*	*D*—H	H⋯*A*	*D*⋯*A*	*D*—H⋯*A*
O3—H3⋯O1^iv^	0.87 (6)	1.87 (7)	2.729 (5)	167 (7)

Symmetry code: (iv) 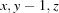.

**Fig. 1.**
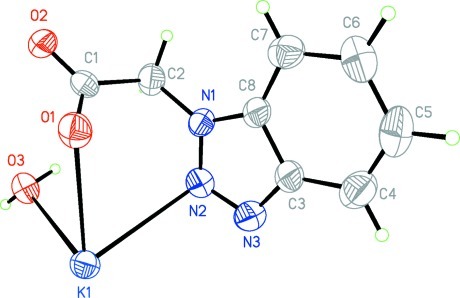
The asymmetric unit of the title complex showing 40% probability displacement ellipsoids for non-hydrogen atoms.

**Fig. 2.**
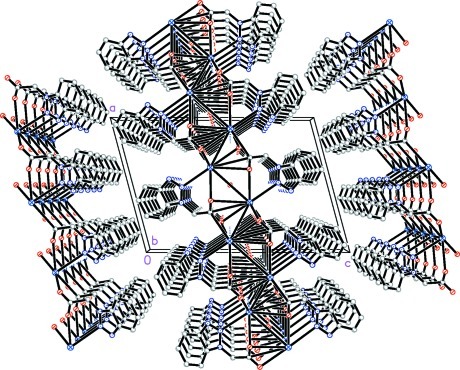
A packing diagram of the title compound, viewed along the b axis, showing the two-dimensional layered structure.

## supplementary crystallographic information

### Comment

Organic ligands based on azole heterocycles or carboxylate groups which contain N and O donors have both good coordination ability and diverse coordination modes (Hu *et al.*, 2008). Therefore, the ligand 1*H*-benzotriazole-1-acetic acid was chosen to create coordination architectures. The synthesis of the ligand was the first step. But, when we synthesized the ligand according to the method of literature (see experimental section), the title complex (I) was obtained instead of the target ligand.

The title complex (I) is a polymeric potassium(I) complex of which the asymmetric is shown in Fig. 1. The environment of the K^I^ ion is a distorted mono-capped octahedral geometry. Each K^I^ ion is coordinated by one O atom from a water molecule, five carboxylate O atoms and one N atom from the ligands. The polymeric structure is a two-dimensional layer parallel to (001) (see, Fig .2).

### Experimental

Reagents and solvents were of commercially available quality. The title compound was synthesized according to the method of Hu *et al.* 2008. To a bromoacetonitrile solution of 1*H*-Benzotriazole (11.9 g), potassium hydroxide (6.8 g), anhydrous K_2_CO_3_ (13.8 g) and TEBA (benzyltriethylammoniumchloride, 99%, 0.15 g) were added. The mixture was stirred at room temperature for 30 min. After cooling to 283 K, 8.4 ml (0.075 mol) ethyl bromoacetate was added dropwise with further stirring. After standing at room temperature overnight the mixture was filtered and the filtrate was distilled under diminished pressure to obtain a yellow liquid. 100 ml water was added to the yellow liquid and the mixture was left to stand for 12 h at a condition of circumfluence. Single crystals suitable for X-ray diffraction were obtained after removing the solvent and recrystallizing in water and methanol solution (30 mL, 5: 1, *v*/*v*) at room temperature. Yield: 20%.

### Refinement

Carbon-bound H-atoms were placed in calculated positions (C—H = 0.93–0.97 Å) and were included in the refinement in the riding model approximation, with *U*_iso_(H) set to 1.2*U*_eq_(C). The unique water H atom was located in a difference Fourier map, and were refined freely.

### Crystal data

**Table:**

[K_2_(C_8_H_6_N_3_O_2_)_2_(H_2_O)]	*F*(000) = 460
*M**_r_* = 448.53	*D*_x_ = 1.564 Mg m^−^^3^
Monoclinic, *C*2	Mo *K*α radiation, λ = 0.71073 Å
Hall symbol: C 2y	Cell parameters from 4308 reflections
*a* = 12.159 (2) Å	θ = 3.4–27.6°
*b* = 4.5893 (9) Å	µ = 0.54 mm^−^^1^
*c* = 17.666 (4) Å	*T* = 296 K
β = 104.98 (3)°	Prism, colorless
*V* = 952.2 (3) Å^3^	0.3 × 0.2 × 0.12 mm
*Z* = 2	

### Data collection

**Table:**

Bruker SMART CCD area-detector diffractometer	2140 independent reflections
Radiation source: fine-focus sealed tube	1508 reflections with *I* > 2σ(*I*)
Graphite monochromator	*R*_int_ = 0.053
φ and ω scans	θ_max_ = 27.5°, θ_min_ = 3.4°
Absorption correction: multi-scan (*SADABS*; Sheldrick, 1996)	*h* = −15→15
*T*_min_ = 0.878, *T*_max_ = 0.937	*k* = −5→5
4827 measured reflections	*l* = −22→22

### Refinement

**Table:**

Refinement on *F*^2^	Secondary atom site location: difference Fourier map
Least-squares matrix: full	Hydrogen site location: inferred from neighbouring sites
*R*[*F*^2^ > 2σ(*F*^2^)] = 0.069	H atoms treated by a mixture of independent and constrained refinement
*wR*(*F*^2^) = 0.135	*w* = 1/[σ^2^(*F*_o_^2^) + (0.0551*P*)^2^] where *P* = (*F*_o_^2^ + 2*F*_c_^2^)/3
*S* = 1.05	(Δ/σ)_max_ < 0.001
2140 reflections	Δρ_max_ = 0.46 e Å^−^^3^
136 parameters	Δρ_min_ = −0.24 e Å^−^^3^
0 restraints	Absolute structure: Flack (1983), 925 Friedel pairs
Primary atom site location: structure-invariant direct methods	Flack parameter: −0.02 (9)

### Special details

**Table:**

Geometry. All e.s.d.'s (except the e.s.d. in the dihedral angle between two l.s. planes) are estimated using the full covariance matrix. The cell e.s.d.'s are taken into account individually in the estimation of e.s.d.'s in distances, angles and torsion angles; correlations between e.s.d.'s in cell parameters are only used when they are defined by crystal symmetry. An approximate (isotropic) treatment of cell e.s.d.'s is used for estimating e.s.d.'s involving l.s. planes.
Refinement. Refinement of *F*^2^ against ALL reflections. The weighted *R*-factor *wR* and goodness of fit *S* are based on *F*^2^, conventional *R*-factors *R* are based on *F*, with *F* set to zero for negative *F*^2^. The threshold expression of *F*^2^ > σ(*F*^2^) is used only for calculating *R*-factors(gt) *etc*. and is not relevant to the choice of reflections for refinement. *R*-factors based on *F*^2^ are statistically about twice as large as those based on *F*, and *R*- factors based on ALL data will be even larger.

### Fractional atomic coordinates and isotropic or equivalent isotropic displacement parameters (Å^2^)

**Table:**

	*x*	*y*	*z*	*U*_iso_*/*U*_eq_	
K1	0.62160 (7)	0.3984 (3)	0.43153 (5)	0.0478 (3)	
O1	0.3919 (3)	0.6125 (7)	0.39356 (19)	0.0563 (9)	
O2	0.2403 (2)	0.3829 (10)	0.41277 (15)	0.0495 (7)	
N1	0.3763 (3)	0.3467 (9)	0.2538 (2)	0.0481 (10)	
N2	0.4845 (3)	0.2466 (10)	0.2740 (2)	0.0601 (12)	
N3	0.5400 (3)	0.3627 (15)	0.2268 (2)	0.0670 (13)	
C1	0.3119 (3)	0.4388 (11)	0.3754 (2)	0.0388 (11)	
C2	0.2979 (4)	0.2654 (12)	0.2991 (3)	0.0541 (13)	
H2A	0.3078	0.0599	0.3119	0.065*	
H2B	0.2208	0.2920	0.2670	0.065*	
C3	0.4673 (4)	0.5401 (12)	0.1754 (3)	0.0489 (12)	
C4	0.4849 (5)	0.7071 (14)	0.1142 (3)	0.0687 (16)	
H4A	0.5550	0.7097	0.1023	0.082*	
C5	0.3952 (5)	0.8677 (19)	0.0721 (3)	0.0718 (16)	
H5A	0.4048	0.9844	0.0313	0.086*	
C6	0.2893 (5)	0.8601 (15)	0.0894 (3)	0.0667 (15)	
H6A	0.2297	0.9691	0.0588	0.080*	
C7	0.2702 (4)	0.6970 (12)	0.1501 (3)	0.0562 (13)	
H7A	0.2001	0.6952	0.1620	0.067*	
C8	0.3618 (4)	0.5360 (11)	0.1923 (3)	0.0442 (11)	
O3	0.5000	0.0180 (11)	0.5000	0.0529 (13)	
H3	0.465 (4)	−0.090 (18)	0.461 (3)	0.083 (17)*	

### Atomic displacement parameters (Å^2^)

**Table:**

	*U*^11^	*U*^22^	*U*^33^	*U*^12^	*U*^13^	*U*^23^
K1	0.0443 (5)	0.0509 (5)	0.0467 (6)	−0.0037 (6)	0.0089 (4)	0.0024 (6)
O1	0.060 (2)	0.054 (2)	0.055 (2)	−0.0158 (18)	0.0148 (16)	−0.0082 (17)
O2	0.0438 (15)	0.0609 (18)	0.0465 (17)	0.003 (2)	0.0165 (12)	0.004 (2)
N1	0.044 (2)	0.062 (3)	0.041 (2)	−0.008 (2)	0.0158 (16)	−0.006 (2)
N2	0.052 (3)	0.081 (3)	0.046 (3)	0.001 (2)	0.0098 (19)	−0.003 (2)
N3	0.050 (2)	0.095 (4)	0.058 (3)	−0.005 (3)	0.017 (2)	−0.008 (3)
C1	0.039 (2)	0.035 (3)	0.038 (2)	0.004 (2)	0.0025 (17)	0.007 (2)
C2	0.057 (3)	0.062 (3)	0.045 (3)	−0.020 (2)	0.016 (2)	−0.007 (2)
C3	0.052 (3)	0.058 (3)	0.037 (3)	−0.010 (3)	0.013 (2)	−0.016 (2)
C4	0.064 (4)	0.085 (4)	0.063 (4)	−0.015 (3)	0.028 (3)	−0.013 (3)
C5	0.103 (4)	0.069 (4)	0.051 (3)	−0.011 (5)	0.032 (3)	−0.003 (4)
C6	0.087 (4)	0.060 (4)	0.050 (3)	0.007 (4)	0.012 (3)	0.002 (3)
C7	0.052 (3)	0.061 (3)	0.057 (3)	0.004 (3)	0.016 (2)	−0.016 (3)
C8	0.045 (3)	0.052 (3)	0.037 (3)	−0.007 (2)	0.013 (2)	−0.017 (2)
O3	0.065 (3)	0.042 (3)	0.048 (3)	0.000	0.008 (3)	0.000

### Geometric parameters (Å, º)

**Table:**

K1—O2^i^	2.718 (4)	C1—C2	1.536 (6)
K1—O3	2.760 (3)	C1—K1^ii^	3.301 (4)
K1—O2^ii^	2.829 (3)	C1—K1^v^	3.461 (5)
K1—O2^iii^	2.835 (4)	C2—H2A	0.9700
K1—O1	2.872 (4)	C2—H2B	0.9700
K1—N2	2.934 (4)	C3—C4	1.386 (7)
K1—O1^ii^	3.287 (3)	C3—C8	1.389 (6)
O1—C1	1.234 (5)	C4—C5	1.366 (8)
O1—K1^ii^	3.287 (3)	C4—H4A	0.9300
O2—C1	1.248 (4)	C5—C6	1.399 (6)
O2—K1^iv^	2.718 (4)	C5—H5A	0.9300
O2—K1^ii^	2.829 (3)	C6—C7	1.376 (7)
O2—K1^v^	2.835 (4)	C6—H6A	0.9300
N1—N2	1.351 (5)	C7—C8	1.383 (7)
N1—C8	1.367 (6)	C7—H7A	0.9300
N1—C2	1.444 (5)	O3—K1^ii^	2.760 (3)
N2—N3	1.314 (6)	O3—H3	0.87 (6)
N3—C3	1.362 (7)		
			
O2^i^—K1—O3	158.80 (10)	O2^i^—K1—H3	171.8 (13)
O2^i^—K1—O2^ii^	86.48 (10)	O3—K1—H3	16.0 (9)
O3—K1—O2^ii^	78.97 (8)	O2^ii^—K1—H3	93.3 (9)
O2^i^—K1—O2^iii^	111.47 (9)	O2^iii^—K1—H3	76.6 (13)
O3—K1—O2^iii^	82.54 (10)	O1—K1—H3	71.3 (12)
O2^ii^—K1—O2^iii^	84.30 (10)	N2—K1—H3	76.2 (9)
O2^i^—K1—O1	101.92 (10)	O1^ii^—K1—H3	82.9 (10)
O3—K1—O1	73.74 (9)	C1^ii^—K1—H3	82.2 (9)
O2^ii^—K1—O1	122.19 (10)	C1^iii^—K1—H3	95.4 (13)
O2^iii^—K1—O1	138.77 (10)	K1^vi^—K1—H3	135.8 (9)
O2^i^—K1—N2	105.55 (11)	K1^vii^—K1—H3	84.5 (11)
O3—K1—N2	91.61 (10)	K1^ii^—K1—H3	49.8 (12)
O2^ii^—K1—N2	164.82 (13)	C1—O1—K1	119.5 (3)
O2^iii^—K1—N2	82.69 (10)	C1—O1—K1^ii^	79.9 (3)
O1—K1—N2	65.15 (10)	K1—O1—K1^ii^	87.70 (10)
O2^i^—K1—O1^ii^	91.55 (9)	C1—O2—K1^iv^	134.2 (4)
O3—K1—O1^ii^	67.34 (9)	C1—O2—K1^ii^	100.9 (2)
O2^ii^—K1—O1^ii^	41.81 (9)	K1^iv^—O2—K1^ii^	95.88 (10)
O2^iii^—K1—O1^ii^	120.67 (8)	C1—O2—K1^v^	109.7 (3)
O1—K1—O1^ii^	80.53 (11)	K1^iv^—O2—K1^v^	111.47 (8)
N2—K1—O1^ii^	143.94 (10)	K1^ii^—O2—K1^v^	93.30 (11)
O2^i^—K1—C1^ii^	94.83 (10)	N2—N1—C8	110.1 (3)
O3—K1—C1^ii^	66.33 (9)	N2—N1—C2	120.2 (4)
O2^ii^—K1—C1^ii^	21.80 (8)	C8—N1—C2	129.6 (4)
O2^iii^—K1—C1^ii^	99.89 (11)	N3—N2—N1	108.6 (4)
O1—K1—C1^ii^	100.70 (11)	N3—N2—K1	104.3 (3)
N2—K1—C1^ii^	157.01 (12)	N1—N2—K1	116.5 (3)
O1^ii^—K1—C1^ii^	21.59 (9)	N2—N3—C3	108.3 (4)
O2^i^—K1—C1^iii^	92.76 (12)	O1—C1—O2	127.2 (4)
O3—K1—C1^iii^	102.35 (12)	O1—C1—C2	117.7 (4)
O2^ii^—K1—C1^iii^	89.46 (10)	O2—C1—C2	115.0 (4)
O2^iii^—K1—C1^iii^	19.86 (9)	O1—C1—K1^ii^	78.5 (2)
O1—K1—C1^iii^	145.45 (10)	O2—C1—K1^ii^	57.28 (19)
N2—K1—C1^iii^	80.89 (11)	C2—C1—K1^ii^	145.0 (3)
O1^ii^—K1—C1^iii^	130.64 (9)	O1—C1—K1^v^	94.1 (3)
C1^ii^—K1—C1^iii^	109.10 (11)	O2—C1—K1^v^	50.5 (3)
O2^i^—K1—K1^vi^	43.10 (5)	C2—C1—K1^v^	129.5 (3)
O3—K1—K1^vi^	120.37 (5)	K1^ii^—C1—K1^v^	75.00 (9)
O2^ii^—K1—K1^vi^	43.41 (9)	N1—C2—C1	114.4 (4)
O2^iii^—K1—K1^vi^	101.65 (7)	N1—C2—H2A	108.7
O1—K1—K1^vi^	119.37 (7)	C1—C2—H2A	108.7
N2—K1—K1^vi^	147.99 (10)	N1—C2—H2B	108.7
O1^ii^—K1—K1^vi^	59.71 (6)	C1—C2—H2B	108.7
C1^ii^—K1—K1^vi^	54.26 (8)	H2A—C2—H2B	107.6
C1^iii^—K1—K1^vi^	92.65 (8)	N3—C3—C4	130.2 (5)
O2^i^—K1—K1^vii^	100.47 (7)	N3—C3—C8	108.9 (4)
O3—K1—K1^vii^	78.52 (5)	C4—C3—C8	120.8 (5)
O2^ii^—K1—K1^vii^	41.03 (8)	C5—C4—C3	117.4 (5)
O2^iii^—K1—K1^vii^	43.29 (5)	C5—C4—H4A	121.3
O1—K1—K1^vii^	150.66 (8)	C3—C4—H4A	121.3
N2—K1—K1^vii^	125.69 (9)	C4—C5—C6	121.2 (6)
O1^ii^—K1—K1^vii^	80.15 (7)	C4—C5—H5A	119.4
C1^ii^—K1—K1^vii^	58.57 (9)	C6—C5—H5A	119.4
C1^iii^—K1—K1^vii^	50.74 (7)	C7—C6—C5	122.3 (6)
K1^vi^—K1—K1^vii^	67.72 (3)	C7—C6—H6A	118.9
O2^i^—K1—K1^ii^	122.38 (7)	C5—C6—H6A	118.9
O3—K1—K1^ii^	39.22 (9)	C6—C7—C8	115.9 (5)
O2^ii^—K1—K1^ii^	76.91 (6)	C6—C7—H7A	122.1
O2^iii^—K1—K1^ii^	120.90 (6)	C8—C7—H7A	122.1
O1—K1—K1^ii^	50.16 (7)	N1—C8—C7	133.5 (4)
N2—K1—K1^ii^	103.29 (9)	N1—C8—C3	104.0 (4)
O1^ii^—K1—K1^ii^	42.14 (6)	C7—C8—C3	122.4 (5)
C1^ii^—K1—K1^ii^	55.66 (8)	K1—O3—K1^ii^	101.56 (17)
C1^iii^—K1—K1^ii^	140.67 (8)	K1—O3—H3	103 (4)
K1^vi^—K1—K1^ii^	101.41 (4)	K1^ii^—O3—H3	120 (4)
K1^vii^—K1—K1^ii^	101.41 (4)		
			
O2^i^—K1—O1—C1	160.5 (3)	K1^ii^—O1—C1—O2	32.1 (5)
O3—K1—O1—C1	−40.9 (3)	K1—O1—C1—C2	−65.4 (4)
O2^ii^—K1—O1—C1	−106.1 (3)	K1^ii^—O1—C1—C2	−146.9 (4)
O2^iii^—K1—O1—C1	16.6 (4)	K1—O1—C1—K1^ii^	81.5 (2)
N2—K1—O1—C1	58.8 (3)	K1—O1—C1—K1^v^	155.27 (17)
O1^ii^—K1—O1—C1	−109.9 (3)	K1^ii^—O1—C1—K1^v^	73.78 (9)
C1^ii^—K1—O1—C1	−102.1 (3)	K1^iv^—O2—C1—O1	−147.7 (4)
C1^iii^—K1—O1—C1	47.4 (4)	K1^ii^—O2—C1—O1	−38.3 (5)
K1^vi^—K1—O1—C1	−157.0 (3)	K1^v^—O2—C1—O1	59.3 (5)
K1^vii^—K1—O1—C1	−60.5 (4)	K1^iv^—O2—C1—C2	31.3 (5)
K1^ii^—K1—O1—C1	−77.0 (3)	K1^ii^—O2—C1—C2	140.8 (3)
O2^i^—K1—O1—K1^ii^	−122.46 (8)	K1^v^—O2—C1—C2	−121.7 (4)
O3—K1—O1—K1^ii^	36.13 (8)	K1^iv^—O2—C1—K1^ii^	−109.5 (3)
O2^ii^—K1—O1—K1^ii^	−29.10 (15)	K1^v^—O2—C1—K1^ii^	97.5 (2)
O2^iii^—K1—O1—K1^ii^	93.58 (13)	K1^iv^—O2—C1—K1^v^	153.0 (4)
N2—K1—O1—K1^ii^	135.76 (13)	K1^ii^—O2—C1—K1^v^	−97.5 (2)
O1^ii^—K1—O1—K1^ii^	−32.88 (11)	N2—N1—C2—C1	79.5 (6)
C1^ii^—K1—O1—K1^ii^	−25.14 (12)	C8—N1—C2—C1	−95.5 (5)
C1^iii^—K1—O1—K1^ii^	124.37 (17)	O1—C1—C2—N1	−4.9 (6)
K1^vi^—K1—O1—K1^ii^	−79.99 (9)	O2—C1—C2—N1	175.9 (4)
K1^vii^—K1—O1—K1^ii^	16.52 (19)	K1^ii^—C1—C2—N1	−116.0 (5)
C8—N1—N2—N3	−0.8 (6)	K1^v^—C1—C2—N1	117.6 (4)
C2—N1—N2—N3	−176.7 (4)	N2—N3—C3—C4	−179.2 (5)
C8—N1—N2—K1	116.5 (3)	N2—N3—C3—C8	0.7 (6)
C2—N1—N2—K1	−59.4 (5)	N3—C3—C4—C5	−179.5 (6)
O2^i^—K1—N2—N3	24.2 (4)	C8—C3—C4—C5	0.6 (7)
O3—K1—N2—N3	−168.4 (4)	C3—C4—C5—C6	−1.0 (9)
O2^ii^—K1—N2—N3	−117.3 (5)	C4—C5—C6—C7	1.4 (10)
O2^iii^—K1—N2—N3	−86.1 (4)	C5—C6—C7—C8	−1.2 (8)
O1—K1—N2—N3	120.4 (4)	N2—N1—C8—C7	−179.7 (5)
O1^ii^—K1—N2—N3	139.7 (3)	C2—N1—C8—C7	−4.3 (9)
C1^ii^—K1—N2—N3	175.8 (4)	N2—N1—C8—C3	1.2 (5)
C1^iii^—K1—N2—N3	−66.1 (4)	C2—N1—C8—C3	176.6 (4)
K1^vi^—K1—N2—N3	14.2 (5)	C6—C7—C8—N1	−178.2 (5)
K1^vii^—K1—N2—N3	−91.4 (4)	C6—C7—C8—C3	0.7 (7)
K1^ii^—K1—N2—N3	153.8 (4)	N3—C3—C8—N1	−1.1 (5)
O2^i^—K1—N2—N1	−95.4 (3)	C4—C3—C8—N1	178.8 (5)
O3—K1—N2—N1	72.0 (3)	N3—C3—C8—C7	179.6 (5)
O2^ii^—K1—N2—N1	123.1 (4)	C4—C3—C8—C7	−0.5 (7)
O2^iii^—K1—N2—N1	154.3 (4)	O2^i^—K1—O3—K1^ii^	35.2 (2)
O1—K1—N2—N1	0.8 (3)	O2^ii^—K1—O3—K1^ii^	82.75 (8)
O1^ii^—K1—N2—N1	20.0 (5)	O2^iii^—K1—O3—K1^ii^	168.36 (6)
C1^ii^—K1—N2—N1	56.2 (5)	O1—K1—O3—K1^ii^	−45.72 (7)
C1^iii^—K1—N2—N1	174.2 (4)	N2—K1—O3—K1^ii^	−109.23 (9)
K1^vi^—K1—N2—N1	−105.4 (3)	O1^ii^—K1—O3—K1^ii^	40.61 (7)
K1^vii^—K1—N2—N1	149.0 (3)	C1^ii^—K1—O3—K1^ii^	64.09 (10)
K1^ii^—K1—N2—N1	34.2 (3)	C1^iii^—K1—O3—K1^ii^	169.75 (7)
N1—N2—N3—C3	0.0 (6)	K1^vi^—K1—O3—K1^ii^	69.19 (5)
K1—N2—N3—C3	−124.8 (4)	K1^vii^—K1—O3—K1^ii^	124.62 (4)
K1—O1—C1—O2	113.6 (5)		

Symmetry codes: (i) *x*+1/2, *y*+1/2, *z*; (ii) −*x*+1, *y*, −*z*+1; (iii) *x*+1/2, *y*−1/2, *z*; (iv) *x*−1/2, *y*−1/2, *z*; (v) *x*−1/2, *y*+1/2, *z*; (vi) −*x*+3/2, *y*+1/2, −*z*+1; (vii) −*x*+3/2, *y*−1/2, −*z*+1.

### Hydrogen-bond geometry (Å, º)

**Table:**

*D*—H···*A*	*D*—H	H···*A*	*D*···*A*	*D*—H···*A*
O3—H3···O1^viii^	0.87 (6)	1.87 (7)	2.729 (5)	167 (7)

Symmetry code: (viii) *x*, *y*−1, *z*.
